# Supplementary material for: Systematic Comparison of the Influence of Different Data Preprocessing Methods on the Performance of Gait Classifications Using Machine Learning
Source: Front Bioeng Biotechnol. 2020 Apr 15;8:260. doi: 10.3389/fbioe.2020.00260 (PMC7174559; doi:10.3389/fbioe.2020.00260)
Supplement: Supplementary file 1 [file Data_Sheet_1.docx]

Supplementary Material

# Supplementary Tables

Supplementary Table S1. Mean **precision**-score for each individual participant depending on each preprocessing method and machine-learning classifier. Each mean value combines all combinations of preprocessing steps where the preprocessing method was part of (n=42).

|  | **GRF**  **filtering** | | **Time**  **derivative** | | **Time**  **normalization** | | | **Data**  **reduction** | | | **Weight**  **normalization** | | **Machine-learning**  **classifier** | | | |
| --- | --- | --- | --- | --- | --- | --- | --- | --- | --- | --- | --- | --- | --- | --- | --- | --- |
|  | **No** | **Yes** | **GRF** | **ΔtGRF** | **11** | **101** | **1001** | **TC** | **TD** | **PCA** | **No** | **Yes** | **SVM** | **RFC** | **MLP** | **CNN** |
| **S01** | 35.2 | 38.8 | 38.2 | 35.8 | 31.7 | 39.3 | 40.0 | 37.0 | 22.9 | 51.1 | 36.7 | 37.3 | 43.2 | 41.8 | 33.4 | 29.6 |
| **S02** | 22.6 | 26.9 | 25.5 | 24.0 | 21.0 | 26.1 | 27.1 | 22.2 | 18.6 | 33.4 | 25.0 | 24.5 | 27.8 | 28.5 | 21.7 | 20.9 |
| **S03** | 33.5 | 40.0 | 37.0 | 36.5 | 32.8 | 40.7 | 36.8 | 37.3 | 24.5 | 48.5 | 36.7 | 36.9 | 41.2 | 40.4 | 35.0 | 30.5 |
| **S04** | 39.6 | 46.9 | 45.0 | 41.8 | 38.4 | 45.4 | 46.2 | 45.6 | 33.5 | 50.1 | 42.6 | 44.0 | 48.0 | 53.0 | 38.8 | 33.5 |
| **S05** | 46.9 | 47.2 | 49.1 | 45.0 | 44.1 | 48.1 | 48.9 | 47.1 | 33.4 | 60.6 | 46.6 | 47.5 | 53.7 | 53.0 | 42.6 | 38.9 |
| **S06** | 36.2 | 37.4 | 37.0 | 36.7 | 29.7 | 40.0 | 41.0 | 36.6 | 25.8 | 47.5 | 37.0 | 36.7 | 40.8 | 42.2 | 36.0 | 28.4 |
| **S07** | 28.3 | 37.0 | 31.8 | 33.4 | 27.3 | 36.5 | 34.1 | 31.2 | 25.6 | 41.1 | 32.8 | 32.5 | 35.8 | 38.2 | 29.5 | 27.0 |
| **S08** | 39.1 | 45.3 | 42.7 | 41.8 | 37.9 | 45.3 | 43.4 | 43.6 | 34.7 | 48.2 | 42.1 | 42.4 | 45.3 | 48.4 | 41.2 | 34.1 |
| **S09** | 40.1 | 44.0 | 42.8 | 41.4 | 36.0 | 46.6 | 43.6 | 40.2 | 31.1 | 54.9 | 42.1 | 42.0 | 48.2 | 46.3 | 38.2 | 35.5 |
| **S10** | 37.9 | 36.9 | 37.9 | 36.9 | 30.9 | 40.7 | 40.6 | 40.9 | 24.4 | 46.9 | 37.0 | 37.8 | 40.4 | 39.5 | 39.0 | 30.6 |
| **S11** | 35.1 | 37.2 | 38.9 | 33.3 | 31.8 | 39.3 | 37.3 | 38.3 | 24.5 | 45.5 | 36.0 | 36.2 | 40.2 | 41.5 | 31.9 | 30.8 |
| **S12** | 31.0 | 28.8 | 33.0 | 26.8 | 25.0 | 32.2 | 32.5 | 32.1 | 20.0 | 37.7 | 30.3 | 29.5 | 33.5 | 31.8 | 31.1 | 23.3 |
| **S13** | 28.6 | 31.1 | 31.1 | 28.6 | 25.5 | 33.5 | 30.5 | 29.3 | 24.7 | 35.6 | 29.6 | 30.2 | 33.6 | 33.2 | 28.2 | 24.5 |
| **S14** | 30.6 | 30.8 | 34.6 | 26.9 | 25.2 | 33.9 | 33.0 | 32.3 | 21.5 | 38.4 | 30.9 | 30.6 | 32.9 | 32.2 | 32.6 | 25.2 |
| **S15** | 36.6 | 41.9 | 43.5 | 35.1 | 33.6 | 43.3 | 41.0 | 39.3 | 28.0 | 50.6 | 39.5 | 39.0 | 45.5 | 42.7 | 36.4 | 32.5 |
| **S16** | 28.7 | 29.6 | 29.5 | 28.8 | 24.5 | 31.1 | 31.8 | 30.5 | 20.4 | 36.6 | 29.4 | 28.9 | 31.0 | 31.2 | 31.4 | 23.1 |
| **S17** | 27.1 | 27.7 | 29.2 | 25.6 | 20.4 | 31.1 | 30.4 | 27.4 | 19.7 | 34.7 | 27.4 | 27.5 | 30.9 | 28.8 | 27.3 | 22.6 |
| **S18** | 22.0 | 24.2 | 23.1 | 23.1 | 20.4 | 24.3 | 24.6 | 25.5 | 15.6 | 28.1 | 23.1 | 23.1 | 25.0 | 24.1 | 24.9 | 18.5 |
| **S19** | 24.5 | 25.9 | 28.5 | 21.8 | 22.3 | 26.5 | 26.7 | 24.0 | 20.1 | 31.4 | 25.1 | 25.2 | 28.2 | 27.6 | 22.8 | 22.1 |
| **S20** | 26.4 | 30.8 | 29.4 | 27.8 | 23.7 | 30.5 | 31.4 | 28.7 | 22.6 | 34.2 | 28.7 | 28.5 | 31.2 | 33.0 | 26.9 | 23.3 |
| **S21** | 25.1 | 26.8 | 28.1 | 23.8 | 22.9 | 28.0 | 26.9 | 25.5 | 19.7 | 32.4 | 25.9 | 26.1 | 28.6 | 30.3 | 23.8 | 21.2 |
| **S22** | 29.1 | 30.6 | 33.3 | 26.4 | 25.7 | 31.4 | 32.5 | 30.8 | 21.4 | 37.3 | 30.2 | 29.6 | 30.5 | 32.1 | 32.6 | 24.2 |
| **S23** | 28.6 | 31.8 | 31.3 | 29.1 | 25.4 | 31.8 | 33.4 | 30.5 | 22.7 | 37.4 | 30.0 | 30.4 | 31.9 | 35.8 | 29.7 | 23.5 |
| **S24** | 32.1 | 39.5 | 36.4 | 35.2 | 30.7 | 37.6 | 39.1 | 36.0 | 28.7 | 42.7 | 36.1 | 35.5 | 39.2 | 40.0 | 35.6 | 28.3 |
| **S25** | 31.4 | 38.5 | 35.8 | 34.0 | 31.4 | 38.1 | 35.2 | 33.8 | 29.7 | 41.3 | 34.9 | 34.9 | 36.6 | 40.2 | 33.7 | 29.2 |
| **S26** | 44.0 | 46.2 | 50.2 | 40.0 | 38.7 | 47.6 | 48.9 | 48.5 | 38.3 | 48.4 | 44.9 | 45.3 | 49.1 | 52.4 | 43.7 | 35.0 |
| **S27** | 28.3 | 28.5 | 27.2 | 29.5 | 23.5 | 30.9 | 30.8 | 30.1 | 21.9 | 33.2 | 28.4 | 28.4 | 29.6 | 31.8 | 28.3 | 23.9 |
| **S28** | 32.6 | 42.3 | 38.5 | 36.4 | 30.4 | 41.8 | 40.2 | 39.6 | 26.9 | 45.9 | 37.7 | 37.3 | 40.4 | 40.3 | 39.2 | 29.9 |
| **S29** | 29.2 | 32.8 | 29.9 | 32.1 | 27.1 | 33.3 | 32.7 | 32.9 | 19.9 | 40.3 | 31.3 | 30.7 | 33.7 | 32.4 | 32.9 | 25.1 |
| **S30** | 28.0 | 29.9 | 32.1 | 25.9 | 25.4 | 29.4 | 32.1 | 31.8 | 18.9 | 35.6 | 28.7 | 29.1 | 30.1 | 34.0 | 29.3 | 22.4 |
| **S31** | 48.7 | 51.4 | 52.2 | 47.9 | 42.1 | 53.5 | 54.5 | 56.3 | 33.9 | 59.9 | 50.0 | 50.1 | 54.5 | 55.6 | 51.4 | 38.7 |
| **S32** | 39.7 | 42.7 | 44.1 | 38.4 | 35.4 | 42.8 | 45.4 | 46.2 | 28.0 | 49.4 | 41.5 | 40.9 | 44.6 | 47.1 | 40.8 | 32.3 |
| **S33** | 32.5 | 38.2 | 36.4 | 34.2 | 29.0 | 37.2 | 39.7 | 38.1 | 20.8 | 46.8 | 34.9 | 35.8 | 39.5 | 38.4 | 36.0 | 27.5 |
| **S34** | 46.6 | 48.6 | 50.6 | 44.5 | 41.3 | 49.9 | 51.4 | 48.8 | 35.7 | 58.2 | 47.5 | 47.7 | 50.7 | 54.2 | 48.2 | 37.2 |
| **S35** | 35.2 | 42.1 | 42.0 | 35.7 | 31.9 | 42.3 | 42.2 | 42.2 | 22.8 | 50.2 | 38.9 | 38.5 | 42.4 | 44.2 | 39.2 | 29.1 |
| **S36** | 33.7 | 36.1 | 37.7 | 32.1 | 29.8 | 37.5 | 37.5 | 36.0 | 26.9 | 41.8 | 34.7 | 35.1 | 37.9 | 39.7 | 33.8 | 28.2 |
| **S37** | 27.8 | 30.5 | 32.5 | 25.9 | 24.6 | 30.4 | 32.6 | 30.4 | 17.6 | 39.4 | 29.0 | 29.3 | 32.2 | 30.0 | 31.1 | 23.4 |
| **S38** | 31.8 | 35.0 | 35.7 | 31.2 | 27.7 | 35.5 | 37.0 | 34.6 | 23.6 | 42.1 | 33.5 | 33.4 | 36.3 | 37.6 | 33.8 | 26.0 |
| **S39** | 38.2 | 39.9 | 42.7 | 35.4 | 35.7 | 39.8 | 41.7 | 43.9 | 25.2 | 48.1 | 39.0 | 39.1 | 40.8 | 45.2 | 39.6 | 30.7 |
| **S40** | 38.1 | 45.6 | 45.4 | 38.3 | 33.9 | 44.6 | 47.0 | 44.7 | 27.1 | 53.6 | 41.9 | 41.9 | 45.7 | 47.0 | 43.7 | 31.1 |
| **S41** | 35.1 | 40.0 | 40.7 | 34.5 | 31.8 | 39.6 | 41.3 | 41.1 | 25.2 | 46.3 | 37.3 | 37.8 | 39.4 | 45.0 | 37.3 | 28.5 |
| **S42** | 24.2 | 26.4 | 28.0 | 22.6 | 22.5 | 25.2 | 28.4 | 26.6 | 18.8 | 29.9 | 25.4 | 25.3 | 26.3 | 28.2 | 25.3 | 21.5 |
| **M** | **33.1** | **36.5** | **36.6** | **33.0** | **29.7** | **37.2** | **37.4** | **36.1** | **24.9** | **43.2** | **34.8** | **34.8** | **38.0** | **39.0** | **34.2** | **27.9** |
| **SD** | **6.5** | **7.1** | **7.2** | **6.5** | **6.1** | **7.1** | **7.1** | **7.6** | **5.3** | **8.2** | **6.6** | **6.8** | **7.6** | **8.1** | **6.6** | **5.1** |

Supplementary Table S2. Mean **recall**-score for each individual participant depending on each preprocessing method and machine-learning classifier. Each mean value combines all combinations of preprocessing steps where the preprocessing method was part of (n=42).

|  | **GRF**  **filtering** | | **Time**  **derivative** | | **Time**  **normalization** | | | **Data**  **reduction** | | | **Weight**  **normalization** | | **Machine-learning**  **classifier** | | | |
| --- | --- | --- | --- | --- | --- | --- | --- | --- | --- | --- | --- | --- | --- | --- | --- | --- |
|  | **No** | **Yes** | **GRF** | **ΔtGRF** | **11** | **101** | **1001** | **TC** | **TD** | **PCA** | **No** | **Yes** | **SVM** | **RFC** | **MLP** | **CNN** |
| **S01** | 46.2 | 50.1 | 49.4 | 46.9 | 42.9 | 50.5 | 51.1 | 48.4 | 34.2 | 61.8 | 47.9 | 48.4 | 53.7 | 52.2 | 45.0 | 41.8 |
| **S02** | 31.3 | 36.6 | 35.1 | 32.9 | 30.3 | 35.5 | 36.2 | 32.0 | 27.8 | 42.2 | 34.2 | 33.8 | 36.5 | 36.8 | 31.8 | 30.8 |
| **S03** | 45.2 | 51.8 | 49.0 | 48.0 | 44.5 | 52.4 | 48.6 | 49.7 | 36.1 | 59.8 | 48.4 | 48.6 | 52.7 | 51.5 | 46.9 | 42.8 |
| **S04** | 51.1 | 57.6 | 56.1 | 52.9 | 49.4 | 56.6 | 57.4 | 56.5 | 44.9 | 61.2 | 53.9 | 55.0 | 58.7 | 63.0 | 50.3 | 45.9 |
| **S05** | 57.7 | 57.5 | 59.3 | 55.8 | 54.8 | 58.6 | 59.4 | 57.9 | 44.9 | 69.9 | 57.0 | 58.2 | 63.5 | 63.1 | 53.5 | 50.2 |
| **S06** | 46.1 | 47.2 | 47.0 | 46.3 | 39.8 | 49.9 | 50.4 | 46.6 | 36.2 | 56.4 | 46.8 | 46.5 | 49.6 | 51.7 | 45.9 | 39.3 |
| **S07** | 39.4 | 48.9 | 43.8 | 44.5 | 38.8 | 47.8 | 45.9 | 43.0 | 36.5 | 53.0 | 44.3 | 44.0 | 46.9 | 49.5 | 41.6 | 38.7 |
| **S08** | 51.1 | 57.2 | 54.9 | 53.5 | 49.9 | 57.2 | 55.3 | 55.7 | 46.9 | 59.7 | 54.1 | 54.3 | 57.0 | 59.9 | 53.4 | 46.5 |
| **S09** | 50.9 | 55.0 | 54.2 | 51.7 | 47.3 | 57.2 | 54.3 | 51.9 | 42.2 | 64.8 | 53.0 | 52.9 | 57.7 | 57.0 | 49.7 | 47.4 |
| **S10** | 49.1 | 48.4 | 48.9 | 48.6 | 42.5 | 51.9 | 51.9 | 52.3 | 35.8 | 58.1 | 48.6 | 48.9 | 51.3 | 51.1 | 50.0 | 42.7 |
| **S11** | 47.2 | 49.4 | 51.2 | 45.4 | 44.4 | 51.2 | 49.2 | 51.0 | 36.1 | 57.8 | 48.2 | 48.4 | 52.4 | 53.0 | 44.4 | 43.4 |
| **S12** | 41.7 | 39.5 | 44.0 | 37.2 | 35.6 | 43.0 | 43.2 | 42.9 | 31.1 | 47.8 | 41.1 | 40.1 | 43.9 | 42.1 | 41.8 | 34.6 |
| **S13** | 39.3 | 42.6 | 42.7 | 39.2 | 36.2 | 44.5 | 42.0 | 40.6 | 35.5 | 46.6 | 40.6 | 41.2 | 44.4 | 44.4 | 39.1 | 35.7 |
| **S14** | 41.8 | 41.7 | 46.2 | 37.4 | 36.2 | 45.2 | 43.9 | 44.0 | 32.1 | 49.2 | 41.9 | 41.7 | 44.1 | 42.9 | 43.5 | 36.7 |
| **S15** | 48.0 | 53.2 | 54.7 | 46.5 | 44.9 | 54.5 | 52.3 | 50.7 | 39.7 | 61.4 | 50.9 | 50.3 | 55.9 | 53.9 | 48.0 | 44.6 |
| **S16** | 40.0 | 41.1 | 41.5 | 39.7 | 35.6 | 42.7 | 43.5 | 41.9 | 31.7 | 48.2 | 40.9 | 40.3 | 42.2 | 42.4 | 42.7 | 35.1 |
| **S17** | 36.5 | 37.0 | 39.1 | 34.4 | 29.9 | 40.4 | 39.7 | 37.7 | 28.8 | 43.4 | 36.8 | 36.7 | 39.5 | 38.3 | 36.9 | 32.4 |
| **S18** | 31.4 | 34.3 | 32.9 | 32.9 | 29.8 | 34.3 | 34.6 | 35.8 | 24.6 | 38.2 | 33.0 | 32.8 | 34.5 | 33.5 | 34.9 | 28.5 |
| **S19** | 34.6 | 35.8 | 38.9 | 31.6 | 32.3 | 36.6 | 36.8 | 34.3 | 30.1 | 41.3 | 35.3 | 35.2 | 37.6 | 37.4 | 33.4 | 32.5 |
| **S20** | 36.9 | 42.1 | 40.2 | 38.8 | 34.3 | 41.4 | 42.6 | 39.6 | 34.1 | 44.6 | 39.6 | 39.3 | 41.7 | 43.9 | 37.9 | 34.4 |
| **S21** | 34.6 | 36.8 | 38.0 | 33.3 | 32.5 | 37.5 | 36.9 | 35.0 | 30.1 | 41.6 | 35.6 | 35.7 | 37.4 | 39.5 | 33.7 | 32.0 |
| **S22** | 40.1 | 40.6 | 44.0 | 36.7 | 36.0 | 41.7 | 43.3 | 42.0 | 31.5 | 47.5 | 40.6 | 40.1 | 40.8 | 42.4 | 43.1 | 35.1 |
| **S23** | 39.4 | 42.6 | 42.6 | 39.4 | 36.5 | 42.4 | 44.0 | 41.7 | 33.2 | 48.0 | 40.7 | 41.2 | 42.0 | 46.5 | 40.6 | 34.8 |
| **S24** | 43.8 | 52.1 | 48.9 | 47.0 | 42.2 | 50.0 | 51.6 | 48.4 | 40.8 | 54.5 | 48.2 | 47.7 | 51.2 | 52.2 | 47.8 | 40.5 |
| **S25** | 42.8 | 50.0 | 47.4 | 45.3 | 42.6 | 49.2 | 47.2 | 45.3 | 41.2 | 52.6 | 46.3 | 46.5 | 48.2 | 51.4 | 45.0 | 40.9 |
| **S26** | 56.0 | 58.3 | 61.6 | 52.7 | 51.2 | 59.7 | 60.5 | 60.9 | 50.4 | 60.1 | 56.9 | 57.4 | 61.1 | 64.5 | 55.0 | 48.0 |
| **S27** | 39.3 | 39.8 | 38.3 | 40.7 | 34.4 | 42.1 | 42.1 | 41.7 | 32.5 | 44.4 | 39.5 | 39.5 | 40.3 | 42.7 | 39.7 | 35.4 |
| **S28** | 43.8 | 53.2 | 50.2 | 46.9 | 41.9 | 52.6 | 51.0 | 50.9 | 38.2 | 56.4 | 48.7 | 48.3 | 50.8 | 51.7 | 50.1 | 41.5 |
| **S29** | 39.6 | 43.8 | 40.9 | 42.5 | 38.0 | 43.9 | 43.2 | 44.2 | 30.0 | 50.9 | 42.0 | 41.4 | 43.9 | 43.1 | 43.6 | 36.3 |
| **S30** | 38.9 | 41.0 | 43.3 | 36.7 | 35.8 | 40.8 | 43.3 | 42.9 | 29.8 | 46.6 | 39.8 | 40.1 | 41.1 | 44.1 | 40.7 | 34.0 |
| **S31** | 58.6 | 60.6 | 61.4 | 57.8 | 52.1 | 62.9 | 63.8 | 65.1 | 45.0 | 68.6 | 59.6 | 59.6 | 63.3 | 65.1 | 60.2 | 49.7 |
| **S32** | 50.7 | 53.6 | 55.1 | 49.2 | 47.0 | 53.3 | 56.1 | 56.9 | 39.5 | 59.9 | 52.4 | 51.9 | 55.2 | 57.3 | 51.9 | 44.2 |
| **S33** | 43.5 | 49.1 | 47.4 | 45.2 | 40.0 | 48.1 | 50.7 | 49.7 | 31.3 | 57.6 | 46.0 | 46.5 | 50.1 | 48.6 | 47.1 | 39.3 |
| **S34** | 57.7 | 59.4 | 61.4 | 55.7 | 52.7 | 60.8 | 62.2 | 59.7 | 47.6 | 68.3 | 58.5 | 58.7 | 61.5 | 64.7 | 59.1 | 48.9 |
| **S35** | 46.5 | 53.4 | 53.3 | 46.8 | 43.6 | 53.4 | 53.3 | 53.6 | 34.5 | 61.0 | 50.2 | 49.8 | 53.2 | 55.1 | 50.4 | 41.3 |
| **S36** | 44.8 | 47.1 | 48.7 | 43.1 | 41.2 | 48.4 | 48.2 | 47.5 | 37.9 | 52.4 | 45.8 | 46.1 | 48.5 | 50.8 | 44.7 | 39.8 |
| **S37** | 38.8 | 41.8 | 43.7 | 36.9 | 36.2 | 41.2 | 43.5 | 41.8 | 28.3 | 50.7 | 40.1 | 40.5 | 42.6 | 41.0 | 42.5 | 35.1 |
| **S38** | 42.8 | 46.1 | 47.1 | 41.9 | 39.3 | 46.3 | 47.9 | 45.5 | 35.0 | 53.0 | 44.6 | 44.4 | 46.8 | 48.3 | 45.2 | 37.6 |
| **S39** | 49.9 | 51.2 | 54.1 | 46.9 | 47.5 | 51.3 | 52.8 | 55.4 | 36.6 | 59.5 | 50.4 | 50.6 | 52.1 | 56.1 | 51.1 | 42.8 |
| **S40** | 49.5 | 56.7 | 56.9 | 49.3 | 45.4 | 55.7 | 58.1 | 55.9 | 39.2 | 64.0 | 53.0 | 53.1 | 56.0 | 58.1 | 54.5 | 43.6 |
| **S41** | 46.5 | 50.9 | 51.8 | 45.6 | 42.5 | 50.9 | 52.7 | 52.3 | 36.8 | 57.0 | 48.5 | 48.9 | 50.0 | 55.4 | 48.8 | 40.6 |
| **S42** | 34.5 | 36.8 | 39.0 | 32.5 | 33.0 | 35.7 | 38.4 | 37.2 | 29.3 | 40.0 | 35.6 | 35.7 | 35.8 | 38.7 | 36.0 | 32.3 |
| **M** | **44.0** | **47.4** | **47.7** | **43.7** | **40.7** | **48.1** | **48.3** | **47.3** | **35.9** | **53.8** | **45.7** | **45.7** | **48.5** | **49.6** | **45.3** | **39.5** |
| **SD** | **6.8** | **7.4** | **7.3** | **6.9** | **6.5** | **7.3** | **7.3** | **7.8** | **5.9** | **8.2** | **6.9** | **7.0** | **7.8** | **8.3** | **6.7** | **5.5** |

*Note*: Since this is a balanced multi-class classification, the recall-score corresponds exactly to the accuracy-score.

Supplementary Table S3. All combinations of data preprocessing methods and machine-learning classifiers, ranked by the mean F1-score over the 15-fold cross validation (n=42).

| **Rank** | **GRF**  **filtering** | **Time**  **derivative** | **Time**  **normalization** | **Data**  **reduction** | **Weight**  **normalization** | **Classifier** | **F1-**  **score** | **Precision** | **Recall** |
| --- | --- | --- | --- | --- | --- | --- | --- | --- | --- |
| 1 | No | GRF | 1001 | PCA | No | SVM | **54.4** | 51.1 | 61.6 |
| 2 | No | GRF | 101 | PCA | Yes | SVM | **54.2** | 51.0 | 61.3 |
| 3 | No | GRF | 1001 | PCA | Yes | SVM | **54.1** | 50.9 | 61.3 |
| 4 | Yes | GRF | 1001 | PCA | No | SVM | **54.0** | 50.9 | 61.1 |
| 5 | Yes | GRF | 101 | PCA | No | SVM | **53.9** | 50.7 | 61.0 |
| 6 | No | GRF | 101 | PCA | No | SVM | **53.8** | 50.5 | 61.1 |
| 7 | Yes | GRF | 1001 | PCA | Yes | SVM | **53.7** | 50.5 | 60.9 |
| 8 | Yes | GRF | 101 | PCA | Yes | SVM | **53.6** | 50.4 | 60.8 |
| 9 | Yes | ΔtGRF | 1001 | PCA | No | SVM | **53.5** | 50.3 | 60.6 |
| 10 | Yes | ΔtGRF | 101 | PCA | No | SVM | **53.2** | 50.1 | 60.3 |
| 11 | Yes | ΔtGRF | 101 | PCA | Yes | SVM | **53.2** | 50.0 | 60.2 |
| 12 | Yes | ΔtGRF | 1001 | PCA | Yes | SVM | **53.2** | 49.9 | 60.4 |
| 13 | No | GRF | 1001 | PCA | No | MLP | **53.0** | 49.8 | 60.1 |
| 14 | Yes | GRF | 101 | PCA | No | MLP | **52.7** | 49.4 | 60.1 |
| 15 | Yes | GRF | 1001 | PCA | No | MLP | **52.7** | 49.4 | 60.0 |
| 16 | No | GRF | 101 | PCA | Yes | MLP | **52.6** | 49.3 | 59.9 |
| 17 | Yes | GRF | 1001 | PCA | Yes | MLP | **52.6** | 49.3 | 59.7 |
| 18 | No | GRF | 1001 | PCA | Yes | MLP | **52.3** | 49.2 | 59.4 |
| 19 | Yes | ΔtGRF | 101 | TC | Yes | RFC | **52.1** | 48.9 | 59.1 |
| 20 | No | GRF | 101 | PCA | No | MLP | **52.1** | 48.7 | 59.5 |
| 21 | Yes | GRF | 101 | PCA | Yes | MLP | **52.1** | 48.7 | 59.4 |
| 22 | Yes | ΔtGRF | 101 | TC | Yes | MLP | **51.6** | 48.5 | 58.6 |
| 23 | Yes | ΔtGRF | 1001 | PCA | Yes | MLP | **51.6** | 48.3 | 58.8 |
| 24 | Yes | ΔtGRF | 101 | PCA | No | MLP | **51.6** | 48.3 | 58.6 |
| 25 | Yes | ΔtGRF | 101 | TC | No | RFC | **51.5** | 48.2 | 58.6 |
| 26 | Yes | ΔtGRF | 1001 | PCA | No | MLP | **51.4** | 48.2 | 58.6 |
| 27 | Yes | ΔtGRF | 1001 | TC | Yes | RFC | **51.4** | 48.0 | 58.8 |
| 28 | Yes | ΔtGRF | 101 | PCA | Yes | MLP | **51.4** | 48.2 | 58.5 |
| 29 | Yes | ΔtGRF | 101 | TC | No | MLP | **51.1** | 47.9 | 58.3 |
| 30 | Yes | ΔtGRF | 1001 | TC | No | RFC | **51.1** | 47.6 | 58.6 |
| 31 | No | ΔtGRF | 1001 | PCA | No | SVM | **50.2** | 47.1 | 57.4 |
| 32 | No | ΔtGRF | 1001 | PCA | Yes | SVM | **50.2** | 47.0 | 57.3 |
| 33 | No | GRF | 101 | TC | Yes | MLP | **49.8** | 46.7 | 57.0 |
| 34 | Yes | ΔtGRF | 1001 | PCA | No | RFC | **49.8** | 46.4 | 57.1 |
| 35 | Yes | ΔtGRF | 1001 | PCA | Yes | RFC | **49.7** | 46.4 | 57.0 |
| 36 | Yes | GRF | 1001 | PCA | Yes | RFC | **49.4** | 46.2 | 56.8 |
| 37 | Yes | GRF | 101 | PCA | No | RFC | **49.3** | 46.0 | 56.7 |
| 38 | Yes | ΔtGRF | 1001 | TC | No | SVM | **49.3** | 46.1 | 56.3 |
| 39 | No | GRF | 1001 | PCA | Yes | RFC | **49.3** | 46.0 | 56.4 |
| 40 | Yes | ΔtGRF | 1001 | TC | Yes | SVM | **49.3** | 46.1 | 56.2 |
| 41 | Yes | ΔtGRF | 101 | PCA | No | RFC | **49.2** | 45.9 | 56.5 |
| 42 | No | GRF | 101 | PCA | No | RFC | **49.2** | 45.8 | 56.6 |
| 43 | Yes | GRF | 1001 | TC | Yes | SVM | **49.1** | 45.8 | 56.3 |
| 44 | Yes | GRF | 1001 | PCA | No | RFC | **49.1** | 45.8 | 56.2 |
| 45 | No | GRF | 1001 | TC | Yes | SVM | **49.0** | 45.8 | 56.2 |
| 46 | Yes | ΔtGRF | 101 | TC | No | SVM | **48.9** | 45.7 | 56.0 |
| 47 | No | GRF | 101 | PCA | Yes | RFC | **48.8** | 45.5 | 56.2 |
| 48 | No | GRF | 101 | TC | No | MLP | **48.7** | 45.6 | 55.8 |
| 49 | Yes | ΔtGRF | 101 | TC | Yes | SVM | **48.7** | 45.6 | 55.8 |
| 50 | Yes | GRF | 101 | PCA | Yes | RFC | **48.7** | 45.4 | 55.8 |
| 51 | No | ΔtGRF | 1001 | TC | Yes | RFC | **48.6** | 45.2 | 56.2 |
| 52 | No | GRF | 1001 | PCA | No | RFC | **48.6** | 45.2 | 56.0 |
| 53 | Yes | ΔtGRF | 101 | PCA | Yes | RFC | **48.5** | 45.1 | 55.9 |
| 54 | Yes | GRF | 1001 | TC | No | SVM | **48.5** | 45.2 | 55.7 |
| 55 | No | GRF | 1001 | TC | No | SVM | **48.3** | 45.1 | 55.5 |
| 56 | Yes | GRF | 101 | TC | Yes | SVM | **48.2** | 44.9 | 55.5 |
| 57 | No | GRF | 101 | PCA | No | CNN | **48.0** | 44.7 | 55.3 |
| 58 | Yes | GRF | 11 | PCA | Yes | SVM | **47.9** | 44.7 | 55.2 |
| 59 | Yes | GRF | 101 | TC | No | SVM | **47.9** | 44.7 | 55.1 |
| 60 | No | ΔtGRF | 1001 | TC | No | RFC | **47.9** | 44.5 | 55.4 |
| 61 | No | GRF | 101 | TC | Yes | SVM | **47.8** | 44.7 | 55.0 |
| 62 | Yes | ΔtGRF | 101 | PCA | Yes | CNN | **47.8** | 44.7 | 54.9 |
| 63 | Yes | GRF | 11 | PCA | No | SVM | **47.7** | 44.5 | 54.9 |
| 64 | No | GRF | 101 | TC | No | SVM | **47.7** | 44.5 | 55.0 |
| 65 | No | ΔtGRF | 1001 | PCA | No | MLP | **47.7** | 44.4 | 55.0 |
| 66 | Yes | GRF | 101 | TC | Yes | MLP | **47.7** | 44.5 | 55.0 |
| 67 | No | ΔtGRF | 1001 | PCA | Yes | MLP | **47.6** | 44.3 | 55.0 |
| 68 | No | GRF | 1001 | PCA | No | CNN | **47.6** | 44.4 | 54.8 |
| 69 | Yes | GRF | 101 | PCA | No | CNN | **47.4** | 44.1 | 54.9 |
| 70 | Yes | GRF | 11 | PCA | No | MLP | **47.4** | 44.1 | 54.6 |
| 71 | Yes | GRF | 1001 | PCA | Yes | CNN | **47.4** | 44.2 | 54.6 |
| 72 | Yes | ΔtGRF | 11 | TC | Yes | RFC | **47.3** | 43.9 | 54.8 |
| 73 | Yes | ΔtGRF | 11 | TC | No | RFC | **47.2** | 43.8 | 54.7 |
| 74 | No | GRF | 11 | PCA | No | SVM | **47.2** | 43.9 | 54.7 |
| 75 | No | GRF | 101 | PCA | Yes | CNN | **47.2** | 43.9 | 54.5 |
| 76 | Yes | GRF | 11 | PCA | Yes | MLP | **47.2** | 43.9 | 54.3 |
| 77 | Yes | GRF | 1001 | PCA | No | CNN | **47.1** | 43.7 | 54.8 |
| 78 | Yes | ΔtGRF | 1001 | PCA | Yes | CNN | **47.1** | 43.8 | 54.5 |
| 79 | No | GRF | 11 | PCA | No | MLP | **46.9** | 43.5 | 54.4 |
| 80 | Yes | GRF | 101 | PCA | Yes | CNN | **46.8** | 43.4 | 54.2 |
| 81 | No | ΔtGRF | 101 | PCA | Yes | SVM | **46.7** | 43.5 | 53.9 |
| 82 | No | GRF | 11 | PCA | Yes | MLP | **46.7** | 43.5 | 54.0 |
| 83 | Yes | ΔtGRF | 101 | PCA | No | CNN | **46.7** | 43.3 | 54.2 |
| 84 | Yes | GRF | 101 | TC | No | MLP | **46.6** | 43.4 | 54.2 |
| 85 | Yes | GRF | 101 | TC | Yes | RFC | **46.6** | 43.2 | 54.2 |
| 86 | Yes | ΔtGRF | 1001 | PCA | No | CNN | **46.6** | 43.4 | 53.8 |
| 87 | No | GRF | 11 | PCA | Yes | SVM | **46.5** | 43.2 | 53.9 |
| 88 | No | ΔtGRF | 101 | PCA | No | SVM | **46.5** | 43.2 | 53.8 |
| 89 | No | GRF | 101 | TC | Yes | RFC | **46.4** | 43.1 | 54.0 |
| 90 | Yes | GRF | 1001 | TC | Yes | RFC | **46.4** | 43.1 | 53.9 |
| 91 | Yes | GRF | 101 | TC | No | RFC | **46.3** | 43.1 | 53.6 |
| 92 | No | GRF | 1001 | PCA | Yes | CNN | **46.1** | 42.9 | 53.3 |
| 93 | Yes | GRF | 1001 | TC | No | RFC | **45.9** | 42.5 | 53.4 |
| 94 | No | GRF | 101 | TC | No | RFC | **45.7** | 42.3 | 53.2 |
| 95 | No | GRF | 1001 | TC | No | RFC | **45.6** | 42.2 | 53.3 |
| 96 | No | ΔtGRF | 101 | TC | Yes | RFC | **45.5** | 42.1 | 53.0 |
| 97 | No | ΔtGRF | 101 | TC | No | RFC | **45.2** | 41.8 | 52.7 |
| 98 | No | GRF | 1001 | TC | Yes | RFC | **45.2** | 41.9 | 52.5 |
| 99 | Yes | GRF | 11 | TC | No | MLP | **45.1** | 41.8 | 52.7 |
| 100 | Yes | GRF | 11 | TC | No | RFC | **45.0** | 41.7 | 52.4 |
| 101 | Yes | GRF | 11 | TC | Yes | MLP | **45.0** | 41.6 | 52.4 |
| 102 | Yes | ΔtGRF | 11 | PCA | Yes | SVM | **44.9** | 41.5 | 52.5 |
| 103 | No | ΔtGRF | 1001 | TC | Yes | SVM | **44.7** | 41.5 | 51.8 |
| 104 | No | ΔtGRF | 1001 | TC | No | SVM | **44.6** | 41.4 | 51.7 |
| 105 | Yes | GRF | 11 | PCA | Yes | RFC | **44.5** | 41.2 | 52.0 |
| 106 | No | GRF | 11 | TC | No | RFC | **44.4** | 41.1 | 51.9 |
| 107 | No | GRF | 11 | TC | Yes | MLP | **44.4** | 41.2 | 51.7 |
| 108 | Yes | GRF | 11 | PCA | No | RFC | **44.4** | 40.9 | 52.0 |
| 109 | No | GRF | 11 | TC | No | MLP | **44.3** | 41.0 | 51.5 |
| 110 | Yes | ΔtGRF | 11 | PCA | No | SVM | **44.3** | 40.9 | 51.9 |
| 111 | Yes | GRF | 11 | TC | Yes | RFC | **44.3** | 41.0 | 51.6 |
| 112 | No | GRF | 11 | PCA | No | RFC | **44.1** | 40.7 | 51.5 |
| 113 | No | GRF | 11 | PCA | Yes | RFC | **43.9** | 40.6 | 51.2 |
| 114 | Yes | GRF | 11 | PCA | Yes | CNN | **43.7** | 40.4 | 51.1 |
| 115 | No | GRF | 11 | TC | Yes | RFC | **43.7** | 40.4 | 51.2 |
| 116 | No | ΔtGRF | 101 | PCA | Yes | MLP | **43.5** | 40.2 | 51.1 |
| 117 | Yes | ΔtGRF | 11 | PCA | Yes | MLP | **43.5** | 40.2 | 51.0 |
| 118 | Yes | ΔtGRF | 11 | PCA | No | MLP | **43.5** | 40.1 | 51.0 |
| 119 | No | ΔtGRF | 101 | PCA | No | MLP | **43.4** | 40.1 | 50.9 |
| 120 | No | ΔtGRF | 1001 | PCA | No | RFC | **43.3** | 39.8 | 51.1 |
| 121 | No | ΔtGRF | 1001 | PCA | Yes | RFC | **43.1** | 39.6 | 50.9 |
| 122 | Yes | GRF | 11 | PCA | No | CNN | **42.8** | 39.5 | 50.3 |
| 123 | Yes | ΔtGRF | 11 | TC | Yes | MLP | **42.5** | 39.3 | 50.0 |
| 124 | Yes | ΔtGRF | 11 | PCA | No | RFC | **42.5** | 39.1 | 50.0 |
| 125 | No | GRF | 11 | PCA | Yes | CNN | **42.4** | 39.1 | 50.0 |
| 126 | No | GRF | 11 | PCA | No | CNN | **42.4** | 39.0 | 50.1 |
| 127 | Yes | ΔtGRF | 11 | TC | No | MLP | **42.4** | 39.2 | 49.6 |
| 128 | Yes | ΔtGRF | 11 | PCA | Yes | RFC | **42.0** | 38.6 | 49.6 |
| 129 | Yes | GRF | 11 | TC | Yes | SVM | **41.9** | 38.7 | 49.1 |
| 130 | Yes | GRF | 11 | TC | No | SVM | **41.7** | 38.6 | 49.0 |
| 131 | No | ΔtGRF | 101 | PCA | Yes | RFC | **41.7** | 38.5 | 49.1 |
| 132 | Yes | ΔtGRF | 11 | PCA | No | CNN | **41.5** | 38.3 | 48.7 |
| 133 | No | ΔtGRF | 101 | PCA | No | RFC | **41.3** | 38.0 | 48.8 |
| 134 | No | GRF | 11 | TC | No | SVM | **40.9** | 37.7 | 48.1 |
| 135 | No | ΔtGRF | 101 | TC | No | SVM | **40.8** | 37.6 | 48.1 |
| 136 | Yes | GRF | 1001 | TD | No | RFC | **40.7** | 37.4 | 48.2 |
| 137 | No | ΔtGRF | 1001 | PCA | Yes | CNN | **40.6** | 37.2 | 48.4 |
| 138 | No | ΔtGRF | 101 | TC | Yes | SVM | **40.5** | 37.3 | 47.9 |
| 139 | Yes | ΔtGRF | 11 | PCA | Yes | CNN | **40.5** | 37.1 | 48.1 |
| 140 | No | GRF | 11 | TC | Yes | SVM | **40.3** | 37.1 | 47.6 |
| 141 | Yes | GRF | 1001 | TD | Yes | RFC | **40.3** | 37.1 | 47.7 |
| 142 | Yes | GRF | 1001 | TD | Yes | MLP | **40.1** | 36.8 | 47.6 |
| 143 | No | GRF | 1001 | TD | Yes | RFC | **39.9** | 36.5 | 47.4 |
| 144 | Yes | ΔtGRF | 101 | TC | No | CNN | **39.8** | 36.4 | 47.7 |
| 145 | Yes | GRF | 1001 | TD | No | MLP | **39.8** | 36.5 | 47.2 |
| 146 | No | ΔtGRF | 1001 | PCA | No | CNN | **39.8** | 36.3 | 47.6 |
| 147 | Yes | GRF | 1001 | TD | Yes | SVM | **39.7** | 36.5 | 47.1 |
| 148 | No | GRF | 1001 | TD | No | RFC | **39.6** | 36.2 | 47.0 |
| 149 | Yes | GRF | 1001 | TD | No | SVM | **39.6** | 36.2 | 47.2 |
| 150 | Yes | ΔtGRF | 101 | TC | Yes | CNN | **39.2** | 35.6 | 47.4 |
| 151 | Yes | GRF | 101 | TD | No | RFC | **38.7** | 35.4 | 46.0 |
| 152 | Yes | GRF | 101 | TD | Yes | MLP | **38.6** | 35.4 | 45.7 |
| 153 | Yes | GRF | 101 | TD | Yes | RFC | **38.5** | 35.2 | 45.9 |
| 154 | Yes | GRF | 101 | TD | No | MLP | **38.4** | 35.1 | 45.8 |
| 155 | No | ΔtGRF | 101 | PCA | Yes | CNN | **38.4** | 35.3 | 45.4 |
| 156 | Yes | ΔtGRF | 11 | TC | Yes | SVM | **38.3** | 35.0 | 45.8 |
| 157 | Yes | ΔtGRF | 11 | TC | No | SVM | **38.3** | 34.9 | 45.9 |
| 158 | No | ΔtGRF | 101 | TC | Yes | MLP | **38.3** | 35.1 | 46.1 |
| 159 | No | ΔtGRF | 101 | TC | No | MLP | **38.1** | 34.9 | 46.1 |
| 160 | Yes | GRF | 1001 | TD | Yes | CNN | **38.0** | 34.7 | 45.6 |
| 161 | Yes | GRF | 101 | TD | Yes | SVM | **37.7** | 34.5 | 45.2 |
| 162 | No | GRF | 1001 | TD | Yes | MLP | **37.7** | 34.5 | 44.8 |
| 163 | No | GRF | 1001 | TD | No | MLP | **37.5** | 34.4 | 44.6 |
| 164 | No | GRF | 1001 | TD | No | CNN | **37.5** | 34.2 | 44.9 |
| 165 | Yes | GRF | 101 | TD | No | SVM | **37.4** | 34.1 | 44.8 |
| 166 | Yes | GRF | 1001 | TD | No | CNN | **37.4** | 34.2 | 44.7 |
| 167 | No | GRF | 1001 | TD | Yes | SVM | **37.4** | 34.2 | 44.6 |
| 168 | Yes | ΔtGRF | 1001 | TD | No | RFC | **37.3** | 34.0 | 44.7 |
| 169 | No | GRF | 1001 | TD | No | SVM | **37.2** | 34.0 | 44.5 |
| 170 | Yes | ΔtGRF | 1001 | TD | Yes | RFC | **37.2** | 34.0 | 44.4 |
| 171 | No | GRF | 1001 | TD | Yes | CNN | **37.0** | 33.9 | 44.2 |
| 172 | Yes | GRF | 101 | TD | No | CNN | **36.9** | 33.9 | 43.9 |
| 173 | Yes | ΔtGRF | 101 | TD | No | RFC | **36.8** | 33.9 | 43.6 |
| 174 | No | ΔtGRF | 101 | PCA | No | CNN | **36.7** | 33.6 | 44.0 |
| 175 | No | GRF | 101 | TD | Yes | RFC | **36.6** | 33.2 | 44.1 |
| 176 | No | GRF | 101 | TD | No | RFC | **36.3** | 33.1 | 43.7 |
| 177 | Yes | GRF | 101 | TD | Yes | CNN | **36.2** | 32.8 | 43.7 |
| 178 | No | ΔtGRF | 11 | TC | Yes | RFC | **36.1** | 33.0 | 43.1 |
| 179 | Yes | ΔtGRF | 101 | TD | Yes | RFC | **36.0** | 33.0 | 43.0 |
| 180 | No | GRF | 11 | TD | Yes | RFC | **36.0** | 32.7 | 43.5 |
| 181 | Yes | ΔtGRF | 1001 | TC | Yes | MLP | **35.8** | 32.8 | 44.8 |
| 182 | No | ΔtGRF | 11 | TC | No | RFC | **35.8** | 32.7 | 42.9 |
| 183 | Yes | GRF | 11 | TD | No | RFC | **35.4** | 32.1 | 42.7 |
| 184 | No | GRF | 11 | TD | No | RFC | **35.3** | 32.1 | 42.8 |
| 185 | Yes | GRF | 11 | TD | Yes | RFC | **35.0** | 31.8 | 42.3 |
| 186 | No | GRF | 101 | TD | No | SVM | **35.0** | 31.9 | 42.4 |
| 187 | No | GRF | 101 | TD | Yes | SVM | **35.0** | 31.8 | 42.5 |
| 188 | No | ΔtGRF | 1001 | TD | No | RFC | **35.0** | 31.9 | 42.0 |
| 189 | Yes | ΔtGRF | 1001 | TC | No | MLP | **34.8** | 31.7 | 43.8 |
| 190 | No | ΔtGRF | 11 | PCA | No | SVM | **34.7** | 31.6 | 41.9 |
| 191 | No | ΔtGRF | 11 | PCA | Yes | SVM | **34.4** | 31.3 | 41.7 |
| 192 | No | ΔtGRF | 11 | PCA | Yes | MLP | **34.3** | 31.2 | 41.3 |
| 193 | No | ΔtGRF | 11 | PCA | No | MLP | **34.2** | 31.2 | 41.1 |
| 194 | No | ΔtGRF | 11 | PCA | No | RFC | **33.9** | 30.7 | 41.3 |
| 195 | No | ΔtGRF | 1001 | TD | Yes | RFC | **33.8** | 30.6 | 41.1 |
| 196 | Yes | ΔtGRF | 11 | TC | No | CNN | **33.6** | 30.5 | 41.0 |
| 197 | No | ΔtGRF | 101 | TC | No | CNN | **33.6** | 30.3 | 41.3 |
| 198 | No | ΔtGRF | 11 | PCA | Yes | RFC | **33.4** | 30.2 | 40.6 |
| 199 | Yes | GRF | 11 | TC | No | CNN | **33.3** | 30.0 | 41.0 |
| 200 | No | GRF | 11 | TC | Yes | CNN | **33.2** | 30.0 | 40.9 |
| 201 | No | ΔtGRF | 11 | TC | No | MLP | **33.2** | 30.2 | 40.4 |
| 202 | No | ΔtGRF | 11 | TC | Yes | MLP | **33.2** | 30.2 | 40.4 |
| 203 | No | ΔtGRF | 101 | TC | Yes | CNN | **33.2** | 30.0 | 40.6 |
| 204 | Yes | ΔtGRF | 11 | TC | Yes | CNN | **33.1** | 29.8 | 40.9 |
| 205 | Yes | GRF | 11 | TC | Yes | CNN | **33.0** | 29.7 | 40.8 |
| 206 | No | GRF | 101 | TD | No | MLP | **32.8** | 29.7 | 40.5 |
| 207 | Yes | ΔtGRF | 11 | TD | Yes | RFC | **32.6** | 29.4 | 39.8 |
| 208 | Yes | ΔtGRF | 1001 | TD | No | MLP | **32.5** | 29.5 | 39.5 |
| 209 | No | ΔtGRF | 1001 | TD | No | SVM | **32.5** | 29.5 | 39.6 |
| 210 | Yes | GRF | 1001 | TC | Yes | MLP | **32.5** | 29.6 | 41.4 |
| 211 | No | ΔtGRF | 1001 | TD | Yes | SVM | **32.4** | 29.5 | 39.3 |
| 212 | Yes | ΔtGRF | 1001 | TD | Yes | MLP | **32.4** | 29.3 | 39.3 |
| 213 | Yes | GRF | 1001 | TC | No | MLP | **32.3** | 29.3 | 41.5 |
| 214 | Yes | ΔtGRF | 11 | TD | No | RFC | **32.3** | 29.1 | 39.5 |
| 215 | No | GRF | 101 | TD | Yes | MLP | **32.2** | 29.2 | 40.1 |
| 216 | No | GRF | 11 | TC | No | CNN | **32.2** | 29.0 | 39.7 |
| 217 | No | ΔtGRF | 11 | PCA | Yes | CNN | **32.1** | 29.2 | 39.0 |
| 218 | No | ΔtGRF | 1001 | TD | No | MLP | **32.0** | 29.1 | 38.9 |
| 219 | No | ΔtGRF | 1001 | TD | Yes | MLP | **31.9** | 28.9 | 38.8 |
| 220 | Yes | ΔtGRF | 1001 | TD | No | SVM | **31.7** | 28.8 | 38.4 |
| 221 | No | GRF | 1001 | TC | No | MLP | **31.6** | 28.8 | 40.8 |
| 222 | Yes | ΔtGRF | 1001 | TD | Yes | CNN | **31.6** | 28.6 | 38.5 |
| 223 | Yes | ΔtGRF | 1001 | TD | Yes | SVM | **31.6** | 28.7 | 38.4 |
| 224 | No | ΔtGRF | 101 | TD | Yes | RFC | **31.6** | 28.6 | 38.4 |
| 225 | No | ΔtGRF | 1001 | TD | No | CNN | **31.5** | 28.4 | 38.7 |
| 226 | No | ΔtGRF | 101 | TD | No | RFC | **31.5** | 28.5 | 38.4 |
| 227 | No | GRF | 1001 | TC | Yes | MLP | **31.3** | 28.4 | 40.3 |
| 228 | Yes | ΔtGRF | 1001 | TD | No | CNN | **31.2** | 28.2 | 38.2 |
| 229 | No | ΔtGRF | 11 | TC | Yes | SVM | **30.9** | 28.0 | 37.8 |
| 230 | No | ΔtGRF | 1001 | TC | No | MLP | **30.7** | 27.9 | 39.7 |
| 231 | No | ΔtGRF | 11 | PCA | No | CNN | **30.5** | 27.5 | 37.5 |
| 232 | No | ΔtGRF | 11 | TC | No | SVM | **30.3** | 27.4 | 37.5 |
| 233 | No | ΔtGRF | 1001 | TD | Yes | CNN | **30.3** | 27.3 | 37.3 |
| 234 | No | ΔtGRF | 1001 | TC | Yes | MLP | **29.9** | 27.1 | 38.9 |
| 235 | No | GRF | 101 | TD | Yes | CNN | **29.8** | 26.9 | 37.9 |
| 236 | No | GRF | 101 | TD | No | CNN | **29.7** | 26.8 | 37.7 |
| 237 | Yes | ΔtGRF | 101 | TD | No | SVM | **29.5** | 26.6 | 36.8 |
| 238 | Yes | ΔtGRF | 101 | TD | Yes | SVM | **29.3** | 26.3 | 36.6 |
| 239 | No | ΔtGRF | 11 | TC | Yes | CNN | **27.7** | 24.8 | 34.8 |
| 240 | No | ΔtGRF | 11 | TC | No | CNN | **27.3** | 24.4 | 34.3 |
| 241 | No | ΔtGRF | 11 | TD | Yes | RFC | **26.7** | 23.8 | 33.5 |
| 242 | No | GRF | 101 | TC | Yes | CNN | **26.5** | 22.4 | 37.4 |
| 243 | Yes | GRF | 101 | TC | No | CNN | **26.2** | 22.2 | 36.9 |
| 244 | Yes | ΔtGRF | 101 | TD | No | MLP | **26.1** | 23.3 | 34.4 |
| 245 | Yes | ΔtGRF | 101 | TD | Yes | MLP | **26.1** | 23.3 | 34.4 |
| 246 | Yes | GRF | 101 | TC | Yes | CNN | **25.9** | 22.0 | 36.6 |
| 247 | Yes | GRF | 11 | TD | Yes | SVM | **25.7** | 22.6 | 33.9 |
| 248 | No | GRF | 101 | TC | No | CNN | **25.5** | 21.5 | 36.4 |
| 249 | No | GRF | 11 | TD | Yes | SVM | **25.3** | 22.2 | 33.7 |
| 250 | No | GRF | 11 | TD | No | SVM | **25.3** | 22.2 | 33.8 |
| 251 | Yes | GRF | 11 | TD | No | SVM | **25.3** | 22.1 | 33.6 |
| 252 | No | ΔtGRF | 11 | TD | No | RFC | **24.8** | 22.0 | 31.4 |
| 253 | No | ΔtGRF | 1001 | TC | Yes | CNN | **24.6** | 21.5 | 34.4 |
| 254 | No | ΔtGRF | 1001 | TC | No | CNN | **24.2** | 21.2 | 34.0 |
| 255 | Yes | ΔtGRF | 11 | TD | No | SVM | **24.1** | 21.2 | 32.0 |
| 256 | Yes | ΔtGRF | 101 | TD | Yes | CNN | **24.1** | 21.4 | 32.4 |
| 257 | Yes | ΔtGRF | 11 | TD | Yes | SVM | **24.0** | 21.0 | 31.9 |
| 258 | Yes | ΔtGRF | 101 | TD | No | CNN | **24.0** | 21.2 | 32.4 |
| 259 | Yes | ΔtGRF | 1001 | TC | No | CNN | **23.6** | 20.5 | 33.7 |
| 260 | Yes | ΔtGRF | 1001 | TC | Yes | CNN | **22.5** | 19.4 | 32.8 |
| 261 | No | ΔtGRF | 101 | TD | Yes | SVM | **21.5** | 18.4 | 29.7 |
| 262 | No | ΔtGRF | 101 | TD | No | SVM | **21.3** | 18.2 | 29.5 |
| 263 | No | ΔtGRF | 11 | TD | Yes | SVM | **18.8** | 16.2 | 25.6 |
| 264 | No | ΔtGRF | 11 | TD | No | SVM | **18.6** | 16.2 | 25.2 |
| 265 | No | GRF | 1001 | TC | Yes | CNN | **15.3** | 12.1 | 26.8 |
| 266 | Yes | GRF | 1001 | TC | Yes | CNN | **14.8** | 11.7 | 26.4 |
| 267 | No | GRF | 1001 | TC | No | CNN | **14.4** | 11.3 | 26.2 |
| 268 | Yes | GRF | 1001 | TC | No | CNN | **13.8** | 10.8 | 25.5 |
| 269 | No | ΔtGRF | 11 | TD | No | MLP | **11.7** | 9.4 | 21.4 |
| 270 | No | ΔtGRF | 11 | TD | No | CNN | **11.7** | 9.4 | 21.3 |
| 271 | No | ΔtGRF | 11 | TD | Yes | MLP | **11.5** | 9.3 | 21.2 |
| 272 | No | ΔtGRF | 11 | TD | Yes | CNN | **11.2** | 9.1 | 20.8 |
| 273 | No | ΔtGRF | 101 | TD | No | MLP | **9.4** | 7.3 | 20.2 |
| 274 | No | ΔtGRF | 101 | TD | Yes | MLP | **9.4** | 7.2 | 20.2 |
| 275 | No | ΔtGRF | 101 | TD | Yes | CNN | **8.8** | 6.7 | 19.6 |
| 276 | No | ΔtGRF | 101 | TD | No | CNN | **8.7** | 6.6 | 19.3 |
| 277 | Yes | ΔtGRF | 11 | TD | No | MLP | **8.2** | 6.1 | 19.3 |
| 278 | Yes | ΔtGRF | 11 | TD | Yes | MLP | **8.2** | 6.1 | 19.3 |
| 279 | Yes | ΔtGRF | 11 | TD | Yes | CNN | **7.9** | 5.7 | 19.0 |
| 280 | Yes | ΔtGRF | 11 | TD | No | CNN | **7.8** | 5.7 | 18.9 |
| 281 | Yes | GRF | 11 | TD | No | MLP | **6.5** | 4.4 | 18.0 |
| 282 | Yes | GRF | 11 | TD | Yes | MLP | **6.5** | 4.4 | 18.0 |
| 283 | Yes | GRF | 11 | TD | No | CNN | **6.4** | 4.3 | 18.0 |
| 284 | Yes | GRF | 11 | TD | Yes | CNN | **6.4** | 4.3 | 17.9 |
| 285 | No | GRF | 11 | TD | No | MLP | **5.6** | 3.6 | 17.4 |
| 286 | No | GRF | 11 | TD | Yes | MLP | **5.6** | 3.6 | 17.2 |
| 287 | No | GRF | 11 | TD | No | CNN | **5.6** | 3.6 | 17.3 |
| 288 | No | GRF | 11 | TD | Yes | CNN | **5.6** | 3.6 | 17.3 |

*Note:* 1) Shown are the rounded mean percentage values of the F1-, precision- and recall-scores; therefore, identical values may occur in the table. However, there are no pairwise identical values, so the ranking is unique. 2) Since this is a balanced multi-class classification, the recall-score corresponds exactly to the accuracy-score.

Supplementary Table S4. Rank scores of all combinations of preprocessing methods depending on their mean **precision**-score over the 15-fold cross validation (n=42).

|  | **GRF**  **filtering** | | **Time**  **derivative** | | **Time**  **normalization** | | | **Data**  **reduction** | | | **Weight**  **normalization** | | **Machine-learning**  **classifier** | | | |
| --- | --- | --- | --- | --- | --- | --- | --- | --- | --- | --- | --- | --- | --- | --- | --- | --- |
|  | **No** | **Yes** | **GRF** | **ΔtGRF** | **11** | **101** | **1001** | **TC** | **TD** | **PCA** | **No** | **Yes** | **SVM** | **RFC** | **MLP** | **CNN** |
| **Score** | 18574 | 22754 | 22945 | 18383 | 10384 | 15506 | 15438 | 14328 | 6884 | 20116 | 20617 | 20711 | 11952 | 12140 | 10396 | 6840 |
| **%**  **max** | 44.2 | 55.8 | 56.3 | 43.7 | 21.1 | 39.6 | 39.3 | 35.3 | 8.4 | 56.3 | 49.9 | 50.1 | 30.2 | 30.8 | 25.2 | 13.8 |

*Note:* The total rank score is for each preprocessing step is 41328. For GRF filtering, time derivative, and weight normalization the minimum rank score is 10296 (0.0%) and the maximum rank score is 31032 (100.0%). For time normalization and data reduction the minimum rank score is 4560 (0.0%) and the maximum is 22992 (66.7%). For the classifiers the minimum rank score is 2556 (0%) and the maximum is 18108 (50.0%). %max: relative rank score of ranks scaled to the interval between the minimum rank score and the maximum total rank score.

Supplementary Table S5. Rank scores of all combinations of preprocessing methods depending on their mean **recall**-score over the 15-fold cross validation (n=42).

|  | **GRF**  **filtering** | | **Time**  **derivative** | | **Time**  **normalization** | | | **Data**  **reduction** | | | **Weight**  **normalization** | | **Machine-learning**  **classifier** | | | |
| --- | --- | --- | --- | --- | --- | --- | --- | --- | --- | --- | --- | --- | --- | --- | --- | --- |
|  | **No** | **Yes** | **GRF** | **ΔtGRF** | **11** | **101** | **1001** | **TC** | **TD** | **PCA** | **No** | **Yes** | **SVM** | **RFC** | **MLP** | **CNN** |
| **Score** | 18542 | 22786 | 23027 | 18301 | 10272 | 15510 | 15546 | 14496 | 6722 | 20110 | 20661 | 20667 | 11830 | 12144 | 10451 | 6903 |
| **%**  **max** | 44.1 | 55.9 | 56.5 | 43.5 | 20.7 | 39.6 | 39.7 | 35.9 | 7.8 | 56.2 | 50.0 | 50.0 | 29.8 | 30.8 | 25.4 | 14.0 |

*Note:* 1) The total rank score is for each preprocessing step is 41328. For GRF filtering, time derivative, and weight normalization the minimum rank score is 10296 (0.0%) and the maximum rank score is 31032 (100.0%). For time normalization and data reduction the minimum rank score is 4560 (0.0%) and the maximum is 22992 (66.7%). For the classifiers the minimum rank score is 2556 (0%) and the maximum is 18108 (50.0%). %max: relative rank score of ranks scaled to the interval between the minimum rank score and the maximum total rank score. 2) Since this is a balanced multi-class classification, the recall-score corresponds exactly to the accuracy-score.
